# Rate-Dependent Hysteresis Modeling and Hybrid Inverse Compensation Control for Piezoelectric Actuators

**DOI:** 10.3390/s26154906

**Published:** 2026-08-03

**Authors:** Qiwei Guo, Zhiliang Yu, Jian Zhou

**Affiliations:** 1 Department of Aeronautics and Astronautics, Fudan University, Shanghai 200437, China; 21110290021@m.fudan.edu.cn; 2 Aerospace System Engineering Shanghai, Shanghai 201100, China; 3 National Key Laboratory of Aerospace Mechanism, Shanghai 201100, China; 4 State Key Laboratory for Strength and Vibration of Mechanical Structures, School of Aerospace Engineering, Xi’an Jiaotong University, Xi’an 710049, China; zhoujian163@xjtu.edu.cn

**Keywords:** piezoelectric actuator, hysteresis modeli-ng, adaptive sliding-mode control, disturbance observer

## Abstract

**Highlights:**

**What are the main findings?**

- A seventh-order, frequency-dependent rising/falling branch model is selected using validation error and endpoint-stability criteria.
- A direct inverse feedforward path is integrated with disturbance-observer-based adaptive sliding-mode feedback and verified on a laboratory piezoelectric stack platform.

**What are the implications of the main findings?**

- The direct inverse model strongly reduces hysteresis nonlinearity, while the feedback loop compensates residual model errors, bounded disturbances, and unmodeled dynamics.
- The hybrid architecture improves tracking beyond feedforward-only compensation while smooth tanh switching and projection adaptation suppress chattering and parameter drift.

**Abstract:**

Piezoelectric ceramic actuators are widely used in precision positioning and sensor-integrated micro-motion systems, but their accuracy is limited by asymmetric, rate-dependent hysteresis and by residual disturbances that remain after feedforward linearization. This study develops a self-contained modeling and control framework that combines an explicit rising/falling branch polynomial model, frequency-dependent coefficient maps, direct inverse feedforward compensation, and disturbance-observer-based adaptive sliding-mode feedback. The actuator is represented as a multilayer piezoelectric stack coupled to an equivalent electrical-mechanical-sensing plant. A branch-state logic resolves the multivalued inverse mapping, and a numerical order-sensitivity study shows that the seventh-order model provides the lowest validation RMSE while avoiding the endpoint growth observed at higher orders. Laboratory measurements at 1, 5, 10, 20, 50, and 100 Hz, together with attenuated, triangular, random-amplitude, step, and 2 Hz sinusoidal tests, are used for validation. The proposed branch model reduces static maximum relative fitting error from 4.50–6.21% for the classical P-I model to 1.28–2.58%. Direct inverse compensation reduces linearity error from 8.56–13.88% to 0.53–1.024%, and the hybrid controller achieves a 1% settling time of 8.6 ms, a maximum tracking error of 0.0051 micrometers, and an RMSE of 0.0012 micrometers. The results demonstrate an embedded-oriented compromise between model accuracy, online computational simplicity, and robust closed-loop precision.

## 1. Introduction

Piezoelectric stack actuators combine high stiffness, rapid electromechanical response, compact dimensions, and sub-micrometer resolution. These characteristics make them central to nano positioning, optical steering, scanning, precision machining, and sensor-integrated micro-motion stages. Their attainable accuracy, however, is governed by the assembled plant rather than by the ceramic element alone; the drive amplifier, compliant transmission, mechanical load, and displacement-sensing chain all contribute dynamics and uncertainty.

Rate-dependent hysteresis is the dominant nonlinear limitation. Wang et al. introduced a dynamic-delay P-I model that represented asymmetric hysteresis from 1 to 250 Hz, but its operator parameters and inverse implementation remain more involved than a direct algebraic mapping [1]. Zhou et al. used separate operator sets for expansion and contraction branches and showed substantial RMSE reductions for asymmetric loops, confirming the value of branch-specific modeling [2].

Data-driven models provide a second route. A high-dimensional Gaussian-process model achieved an NRM-SE of 1.0759% for a four-frequency mixed signal, but it requires multi-dimensional temporal features and Gaussian-process evaluation [3]. A recurrent neural-network inverse reduced the maximum 10 Hz displacement error from 8.96 to 0.465 micrometers, although training data, network storage, and online inference increase implementation requirements [4].

Recent control studies have therefore combined feedforward hysteresis compensation with feedback. Modified Preisach plus robust H-infinity control, ANN-based model-predictive control, phase-hysteresis feedforward-feedback control, and neural feedforward with terminal sliding-mode feedback all improve robustness to model mismatch and disturbances [5,6,7,8]. These approaches demonstrate the benefit of hybrid control, but they do not simultaneously provide an explicit branch polynomial, frequency-coefficient mapping, direct branchwise inverse identification, and disturbance-observer-based adaptive feedback in one embedded-oriented formulation [9,10,11,12,13].

The research gap addressed here is the absence of a compact and transparent modeling-compensation-control chain that (i) represents asymmetric and rate-dependent hysteresis with algebraic expressions, (ii) resolves the multivalued inverse mapping through explicit branch-state logic, (iii) quantifies polynomial-order selection and endpoint robustness, and (iv) experimentally separates model-prediction error from residual closed-loop tracking error [14,15,16,17,18].

The scientific novelty is fourfold. First, the improved P-I integral representation is converted into separate rising and falling polynomials whose coefficients vary quadratically with excitation frequency [19,20,21]. Second, the inverse is identified directly on branch-separated laboratory data, eliminating online algebraic inversion and defining deterministic switching at turning points [22,23,24,25,26,27,28,29]. Third, a realizable disturbance observer and adaptive sliding-mode feedback compensate residual hysteresis, plant uncertainty, and external disturbance after feedforward linearization. Fourth, the model and controller are evaluated with fixed-frequency, attenuated, triangular, random-amplitude, step, and sinusoidal laboratory trajectories, and the validated frequency claim is explicitly limited to 1–100 Hz.

Accordingly, this manuscript is a single, self-contained study rather than a presentation of a second research part. Section 2 describes the actuator structure, electromechanical model, branch identification, inverse logic, and hybrid controller. Section 3 reports numerical order sensitivity and laboratory model/compensation/control results. Section 4 compares the findings with recent operator-based and data-driven approaches and defines the validated scope [30,31,32,33].

The objective is to deliver a reproducible, embedded-oriented method in which the inverse model removes the dominant hysteresis and the feedback loop corrects the smaller residual error without requiring a computationally intensive online hysteresis operator or neural network [34,35,36,37].

## 2. Compensation Methods

### 2.1. Sensor-Integrated Piezoelectric Actuator Structure and Platform

**Figure 1:**
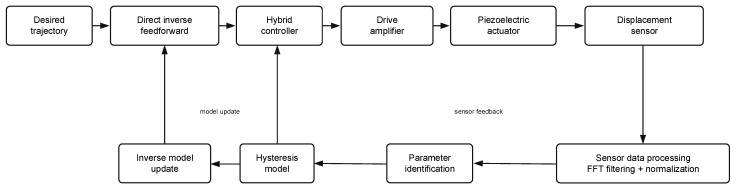
Sensor-integrated architecture for hysteresis modeling, parameter identification, inverse compensation, and hybrid feedback control of a piezoelectric actuator.

The tested device is a sensor-integrated multilayer piezoelectric stack actuator. Alternating internal electrodes divide the ceramic into thin active layers, so the applied voltage produces axial strain through the piezoelectric effect. End caps and mechanical preload maintain compressive loading, while an integrated strain/displacement sensing channel measures the axial motion. The stack drives a compliant or flexible-hinge load through a high-voltage amplifier. Figure 1 summarizes the actuator structure andshows the identification and hybrid-control signal path used throughout the study.

For control design, the platform is decomposed into a nonlinear hysteresis block surrounded by approximately linear electrical, mechanical, and sensing subsystems. The same actuator and sensing chain are used for the hysteresis curves in Figure 1. This consistent hardware path ensures that the numerical model predictions are compared with laboratory sensor measurements rather than with a separate simulated plant.

### 2.2. Classical Prandtl–Ishlinskii Model and Main Limitations

In the classical discrete P-I formulation, the actuator displacement is represented by a weighted superposition of play operators. Let u(t) denote input voltage, y(t) denote actuator displacement, q denote a linear gain, $p_{i}$ denote the operator weight, and $F_{r_{i}}[u](t)$ denote the play operator with threshold $r_{i}$. The discrete model is written as

(1) $$y(t)=qu(t)+\sum_{i=1}^{N}p_{i}F_{r_{i}}[u](t)$$

Although the expression is compact, it has three practical limitations for embedded precision positioning. First, the classical play operator is rate independent and symmetric, so it cannot directly describe asymmetric or frequency-dependent hysteresis. Second, the fitting accuracy is sensitive to the number and distribution of operators. Third, the operator-based implementation increases computational burden when the model is deployed on resource-constrained controllers. These limitations motivate the branch polynomial formulation used in this work.

### 2.3. Modified Static Branch Model

The hysteresis loop is divided into a rising branch and a descending branch. The rising branch corresponds to du/dt ≥ 0 and the descending branch corresponds to du/dt ≤ 0. After the integral density function in the improved P-I derivation is selected and the integral operation is eliminated, each branch can be represented by an explicit polynomial expression. For branch s in {r,d}, the static branch model is expressed as

(2) $$y_{s}(u)=a_{s,0}+a_{s,1}u+a_{s,2}u^{2}+\cdots +a_{s,n_{s}}u^{n_{s}}+b_{s}u+c_{s}$$

The branch-dependent order is selected by minimizing validation error while constraining endpoint behavior. In addition to the least-squares criterion in Equation (3), the implementation checks training RMSE, validation RMSE, and the maximum deviation in a mild extrapolation interval u/Umax in [−0.05, 1.05]. The order-sensitivity calculation in Table 1 uses the identified 1 Hz rising and falling branch curves, 201 uniformly sampled points per branch, additive zero-mean normalized noise with standard deviation 0.001, a 70/30 training-validation split, and 100 Monte Carlo repetitions. The seventh-order polynomial gives the lowest mean validation RMSE; orders above seven reduce training error only marginally but increase endpoint deviation, which is undesirable for an embedded inverse model in Table 1.

(3) $$\left\{\begin{array}{l} E(n)=\sum_{k=1}^{M}{\left[y_{k}-{\hat{y}}_{k}(n)\right]}^{2}\\ n^{*}=\arg\min_{n}E(n) \end{array}\right.\;$$

The initial loading curve is treated separately because it does not repeat in subsequent closed hysteresis loops and does not satisfy congruency. A segmented exponential approximation is used for this initial stage, while the repeated closed loops are described by the branch polynomial model.

### 2.4. Frequency-Dependent Dynamic Hysteresis Model

**Figure 2:**
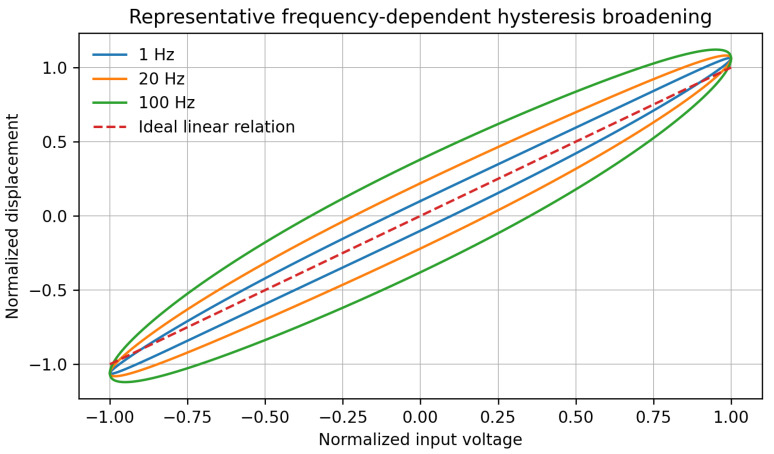
Frequency-dependent hysteresis of the tested sensor-integrated multi-layer stack actuator. Voltage and displacement are normalized by Umax and Ymax; both axes are dimensionless.

Dynamic hysteresis is introduced by making the rising and falling polynomial coefficients depend on excitation frequency f. Figure 2 identifies the response as that of the same sensor-integrated multilayer stack is described. The horizontal coordinate is u/Umax and the vertical coordinate is y/Ymax; both are dimensionless normalized quantities. The widening loop with frequency motivates the coefficient-frequency mapping:

(4) $$a_{s,j}(f)=\alpha_{s,j,0}+\alpha_{s,j,1}f+\alpha_{s,j,2}f^{2}$$

(5) $$y_{s}(u,f)=\sum_{j=0}^{n_{s}}a_{s,j}(f)u^{j}$$

This representation captures the experimentally observed broadening of the hysteresis loop as input frequency increases. More importantly, it keeps the online computation algebraic: once the frequency is known or estimated, the controller evaluates a polynomial rather than a set of play operators.

### 2.5. Parameter Identification and Signal Preprocessing

**Figure 3:**
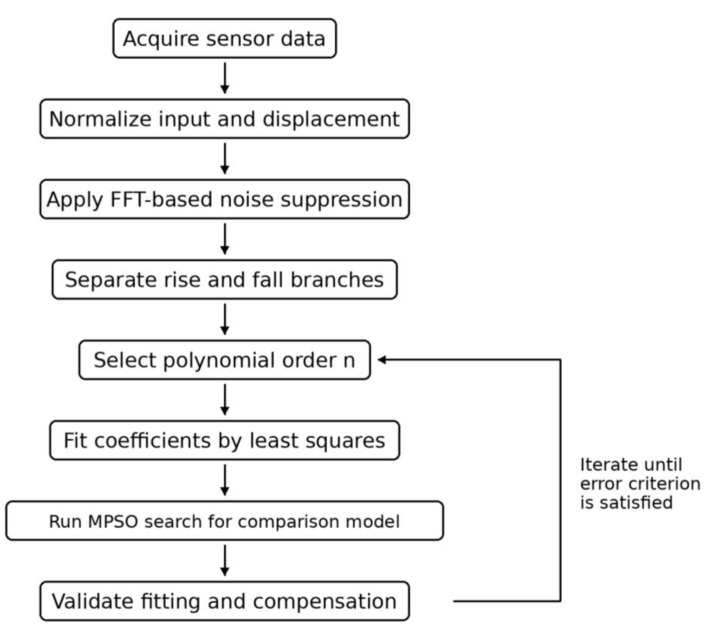
Identification and validation workflow, including turning-point detection, branch separation, polynomial-order validation, frequency mapping, and direct inverse identification.

The identification procedure uses synchronized voltage-displacement records from the sensor channel. Figure 3 summarizes the complete preprocessing and validation work-flow. After normalization and FFT-based noise suppression, turning points are detected from the sign of the filtered input increment. Rising and falling samples are stored separately, candidate orders are fitted by least squares, and the selected branch model is mapped to frequency. The classical P-I comparison model is identified by modified particle swarm optimization using the same laboratory records.

The modified particle swarm optimization algorithm uses a nonlinear decreasing inertia weight and an additional random historical-best particle term. This modification helps reduce premature convergence and improves the balance between global exploration and local search. The particle velocity update can be summarized as

(6) $$v_{i}^{k+1}=\omega_{k}v_{i}^{k}+c_{1}r_{1}\left(p_{i}^{k}-x_{i}^{k}\right)+c_{2}r_{2}\left(g^{k}-x_{i}^{k}\right)+c_{3}r_{3}\left(q^{k}-x_{i}^{k}\right)$$

(7) $$\omega_{k}=\omega_{\min}\exp\left[\frac{\omega_{\max}-\omega_{\min}}{T}(T-k)\right]$$

where $p_{i}^{k}$ is the personal best position, $g^{k}$ is the global best position, $q^{k}$ is a randomly selected historical best position, $c_{1}c_{2}c_{3}$ are acceleration coefficients, $r_{1}r_{2}r_{3}$ are random variables in [0, 1], and T is the maximum iteration number.

### 2.6. Direct Inverse Feedforward Compensation

Instead of algebraically inverting the positive high-order model online, the inverse mapping is identified directly from branch-separated data. Let $x_{d}$ be the desired normalized displacement and $u_{ff,s}$ the feedforward voltage for branch s. The multivalued inverse is made single-valued by retaining a branch-state variable: the rising inverse is selected when Delta $x_{d}$ > delta, the falling inverse when Delta $x_{d}$ < delta, and the previous branch is retained inside the deadband |Delta $x_{d}$| ≤ delta. At a detected turning point, the branch state and last reversal displacement are updated before evaluating Equation (8)

(8) $$u_{ff,s}(x_{d},f)=\sum_{j=0}^{m_{s}}\theta_{s,j}(f)x_{d}^{j}$$

During preprocessing, filtered samples are scanned sequentially; zero-crossings of the displacement increment identify reversal neighborhoods, and a minimum-duration rule rejects noise-induced false switch. Each accepted segment is checked for monotonicity, sorted by displacement, and fitted independently. This branch-state logic resolves the inverse multivalue problem without choosing between two voltages solely from the current displacement. Small local discontinuities caused by sampling delay or sensor noise are corrected only within the reversal neighborhood, preserving global monotonicity. The full algorithm is included in Figure 3.

### 2.7. Integrated Electrical-Mechanical Platform Model

**Figure 4:**
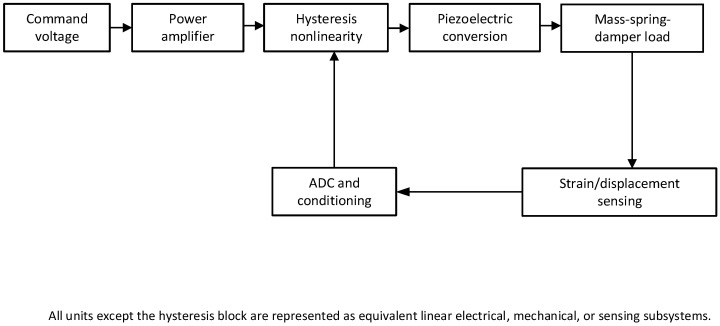
Integrated electrical-mechanical model of the piezoelectric platform. The hysteresis block is treated as the main nonlinear element, while the surrounding electrical, mechanical, and sensing units are represented by equivalent linear subsystems.

The actuator-level hysteresis model is embedded in the complete electrical-mechanical-sensing plant shown in Figure 4. The electrical unit contains the drive amplifier and equivalent resistance-capacitance dynamics; the mechanical unit is a mass-spring-damper representation of the stack, compliant transmission, and moving stage; and the sensor unit includes strain/displacement conversion, conditioning, and digitization.

(9) $$G_{e}(s)=\frac{K_{a}K_{e}}{RCs+1}$$

(10) $$G_{m}(s)=\frac{K_{m}}{ms^{2}+cs+k}$$

(11) G_p(s)=G_e(s)G_m(s)G_s(s)

Here $K_{a}K_{e}$ is the amplifier gain, *R* and *C* are equivalent circuit parameters, $K_{m}$ is an equivalent piezoelectric-mechanical gain, m is the equivalent mass, c is the damping coefficient, k is stiffness, and G_s(s) represents the sensing and conditioning chain. In the controller design stage, the high-order comprehensive model is simplified to a second-order nominal plant because the third-order term in the identified transfer function is small compared with the lower-order terms.

### 2.8. Disturbance Observer for Residual Hysteresis and External Disturbance

**Figure 5:**
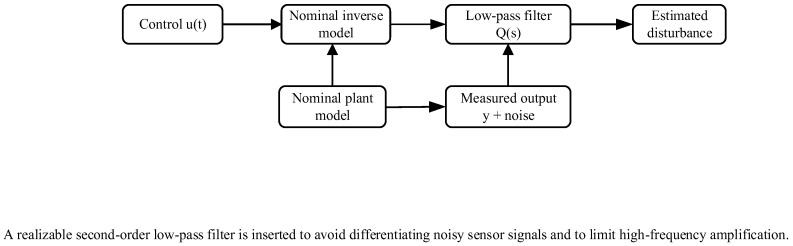
Realizable disturbance observer using a nominal inverse model and a second-order low-pass filter to avoid differentiating noisy sensor signals.

After direct inverse compensation, the remaining nonlinearities are collected with unmodeled dynamics, temperature drift, preload variation, electromagnetic interference, and measurement uncertainty into a bounded disturbance. The realizable observer arrangement is shown in Figure 5. The simplified controlled plant is written as

(12) $$\ddot{y}=-a_{1}\dot{y}-a_{0}y+bu+d(t)$$

where y is platform displacement, u is the mixed control voltage after feedforward and feedback summation, $a_{0}$, $a_{1}$, and *b* are nominal plant parameters, and *d*(t) is the lumped disturbance. A disturbance observer estimates *d*(t) so that it can be compensated in the control law. Direct inversion of the plant is not physically realizable and would amplify sensor noise; therefore, a second-order low-pass filter is inserted in the observer path:

(13) $$Q(s)=\frac{\omega_{q}^{2}}{s^{2}+2\zeta_{q}\omega_{q}s+\omega_{q}^{2}}$$

The cut-off frequency $\omega_{q}$ and damping ratio $\zeta_{q}$ are selected to balance disturbance-rejection bandwidth and noise sensitivity. A larger bandwidth improves disturbance estimation but may amplify high-frequency sensor noise; a smaller bandwidth improves robustness but slows disturbance compensation in Figure 5.

### 2.9. Adaptive Sliding-Mode Feedback Controller

**Figure 6:**
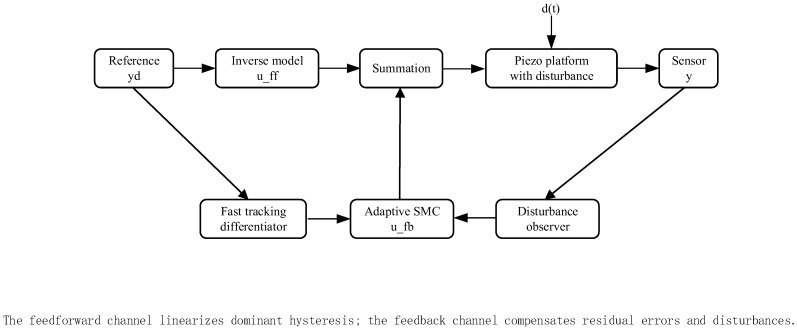
Hybrid control architecture combining direct inverse feedforward compensation, adaptive sliding-mode feedback, disturbance observation, and fast nonlinear tracking differentiation.

The feedforward channel provides the main hysteresis linearization, while the feedback channel corrects residual errors. The resulting signal routing is shown in Figure 6. Let $y_{d}$ be the desired displacement, $e=y-y_{d}$ be tracking error, and λ>0 be a sliding-surface coefficient. The sliding surface is defined as

(14) $$s=\dot{e}+\lambda e$$

For the second-order plant in Equation (12), a baseline adaptive sliding-mode control law can be expressed as

(15) $$u_{fb}={\hat{b}}^{-1}\left[{\ddot{y}}_{d}-{\hat{a}}_{1}\dot{y}-{\hat{a}}_{0}y-\lambda \dot{e}-k_{s}s-\rho \mathrm{sign}(s)-\hat{d}\right]$$

where $\hat{d}$ is the disturbance-observer estimate, $k_{s}$ and ρ are positive switching gains, ${\hat{a}}_{0}$ and ${\hat{a}}_{1}$ and $\hat{b}$ denote estimated plant parameters. The adaptive law updates the unknown coefficients so that tracking remains robust when the identified model differs from the actual platform.

(16) $$\dot{\hat{\theta }}=\Gamma \varphi \left(y,\dot{y},y_{d},{\dot{y}}_{d},{\ddot{y}}_{d}\right)s$$

A Lyapunov candidate containing both the sliding variable and parameter-estimation error can be used to prove that the closed-loop system is stable and that the tracking error converges to a bounded neighborhood of the origin under bounded disturbance.

### 2.10. Improved Smooth Switching Law and Projection Adaptation

The discontinuous sign term in a standard sliding-mode controller can generate chattering in a real piezoelectric drive system. Chattering is undesirable because it excites unmodeled mechanical dynamics, increases amplifier stress, and may contaminate the sensor measurement. The improved controller replaces sign(s) with a smooth hyperbolic tangent function and uses projection adaptation to avoid unbounded parameter growth:

(17) $$u_{fb}={\hat{b}}^{-1}\left[{\ddot{y}}_{d}-{\hat{a}}_{1}\dot{y}-{\hat{a}}_{0}y-\lambda \dot{e}-k_{s}s-\rho \tanh\left(\frac{s}{\phi }\right)-\hat{d}\right]$$

(18) $$\dot{\hat{\theta }}=\mathrm{Proj}\left(\hat{\theta },\Gamma \varphi s\right)$$

The (18) projection operator is introduced into the adaptive law to guarantee the boundedness of the estimated parameters. When the parameter estimates lie inside the prescribed compact set, the adaptive law is equivalent to the conventional update law. When the estimate reaches the boundary and tends to move outward, the projection operator modifies the update direction to prevent parameter drift.

The boundary-layer parameter ϕ controls the smoothness of switching. A small ϕ approximates the sign function and improves convergence speed but may increase switching activity; a larger ϕ improves smoothness but may enlarge the steady-state error bound. The projection operator keeps parameter estimates within a physically meaningful range obtained from calculation, calibration, or preliminary experiments in Figure 6.

### 2.11. Sliding Mode Design

Sliding-mode design requires derivative information, but practical piezoelectric platforms usually do not have direct sensors for all derivative signals. A fast nonlinear tracking differentiator is introduced to generate smooth estimates of the reference signal and its derivatives. The general differentiator form is

(19) $${\dot{z}}_{1}=z_{2},\;{\dot{z}}_{2}=F\left(z_{1}-v,z_{2},r,h\right)$$

where v is the input signal, $z_{1}$ tracks $z_{2}$ estimates its derivative, r controls convergence speed, and h is a filtering or discretization parameter. To improve robustness to measurement noise and modeling error, the differentiator is preceded or accompanied by a second-order filter similar to Equation (13). This design reduces phase lag while avoiding direct differentiation of noisy sensor measurements.

### 2.12. Laboratory Verification Protocol

All model and controller performance indices reported in Section 3 are obtained by comparing numerical predictions or commanded trajectories with the displacement measured by the sensor-integrated actuator described in Section 2.1. Fixed-frequency sinusoidal records at 1, 5, 10, 20, 50, and 100 Hz are used to identify the frequency-coefficient maps. Attenuated sine and triangular signals and random-amplitude sine and triangular signals are reserved for independent validation; the triangular trajectories explicitly test non-smooth reversal points.

The controller is verified in three laboratory tests: a sign-versus-tanh switching comparison, a 1 micrometer step response, and a 2 Hz sinusoidal tracking test. The metrics are maximum absolute error, maximum relative error, RMSE, 1% settling time, and control-input smoothness. Modeling figures use normalized dimensionless axes, whereas the step and tracking figures report calibrated displacement in micrometers.

The validated frequency range of the coefficient map is 1–100 Hz, which corresponds to the intended operating band of the present platform. No performance claim is made above 100 Hz. Higher-frequency validation would require a separate identification of amplifier and stage dynamics; this limitation is stated explicitly rather than extrapolating the quadratic frequency map beyond the measured range.

#### 2.12.1. Complete Platform Decomposition

**Figure 7:**
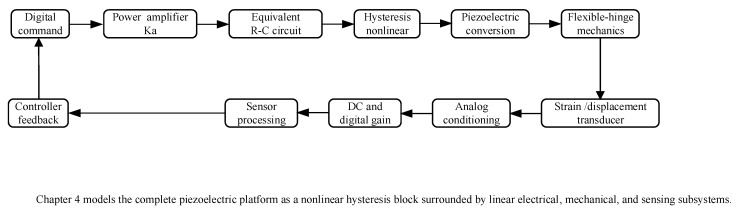
The complete drive system includes power amplification, equivalent electrical dynamics, actuator hysteresis, piezoelectric conversion, flexible-hinge mechanics, displacement or strain sensing, signal conditioning, ADC conversion, and feedback preprocessing.

The full piezoelectric drive platform is decomposed into four functionally different modules. The first is the electrical driving unit, which receives the digital command and produces the high-voltage excitation required by the piezoelectric ceramic. The second is the hysteresis and piezoelectric-conversion unit, where the dominant nonlinear memory effect appears. The third is the mechanical transmission unit, represented by a flexible hinge, rolling contact, rigid platform, and equivalent mass-spring-damper dynamics. The fourth is the sensor and signal-conditioning unit, which converts deformation into a voltage, amplifies it, digitizes it, and feeds it back to the controller in Figure 7.

The reference chapter emphasizes that only the hysteresis block is treated as the principal nonlinear element. The amplifier, equivalent resistance-capacitance circuit, sensing amplifier, analog-to-digital converter, flexible hinge, and rigid stage are modeled as linear or locally linearized subsystems. This decomposition is important because it allows the polynomial inverse compensator to focus on hysteresis, while robust feedback handles the residual mismatch of the complete platform.

#### 2.12.2. Electrical Drive and Detection Models

The electrical unit is modeled from the viewpoint of signal transmission. A voltage command is amplified by the drive circuit and filtered by the equivalent resistance-capacitance dynamics before it excites the piezoelectric stack. Let $K_{a}$ denote the amplifier gain, R and C denote the equivalent resistance and capacitance, and $K_{e}$ denote the equivalent electromechanical conversion coefficient. A compact representation of the electrical drive path is

(20) $$G_{e}(s)=\frac{K_{a}K_{e}}{RCs+1}$$

The detection model is equally important because the controller uses measured displacement rather than the true internal deformation. In the reference platform, the deformation signal is obtained by an internal strain element and a deformation-to-voltage conversion circuit. Because the raw voltage is small and easily contaminated by disturbance, an external detection amplifier and digital conversion chain are introduced. The detection path can be represented as a gain and conditioning block.

(21) y_m(s)=K_sK_{ADC}G_f(s)y(s)+n(s)

where y_m(s) is the measured signal, y is the actual displacement, K_s is the deformation-to-voltage coefficient, K_{ADC} is the digital conversion/scaling coefficient, G_f(s) represents the conditioning filter, and n(s) is measurement noise. This equation clarifies why controller design must avoid direct differentiation of sensor signals and why the disturbance observer requires low-pass filtering.

#### 2.12.3. Flexible-Hinge Mechanical Dynamics

The piezoelectric ceramic itself cannot usually drive the load directly in a precision platform. The actuator is coupled to a flexible hinge and a rigid moving stage, and the mechanical portion can be reduced to a mass-spring-damper model. Using Newtonian mechanics, the mechanical equilibrium is written as

(22) $$m\ddot{y}(t)+c\dot{y}(t)+ky(t)=F_{p}(t)-F_{L}(t)$$

where m is equivalent mass, c is damping, k is stiffness, $F_{p}$ is the piezoelectric driving force, and $F_{L}$ is the external load or disturbance force. In the reference design, the control analysis focuses on the intrinsic hysteresis and tracking performance, so the external load term is first neglected for nominal identification and later collected into the lumped disturbance term for robust control.

(23) $$G_{m}(s)=\frac{Y(s)}{F_{p}(s)}=\frac{1}{ms^{2}+cs+k}$$

Combining the electrical driving path, hysteresis compensation path, piezoelectric conversion, mechanical path, and sensing path yields an integrated model. The reference chapter notes that the identified comprehensive model can be expressed as a third-order linear system cascaded with a nonlinear hysteresis element, but the cubic term is small enough for controller design to use a second-order nominal model.

(24) $$G_{nom}(s)=\frac{b}{s^{2}+a_{1}s+a_{0}}$$

#### 2.12.4. Model Identification from Step Excitation

The reference chapter identifies equivalent parameters that cannot be obtained directly from geometry or circuit design, including equivalent resistance, capacitance, and piezoelectric conversion parameters. A step excitation is applied to the platform, transient input-output data are collected, and the equivalent parameters are fitted. Parameters that are fixed by circuit design, such as drive gain and detection gain, are treated as known constants. Parameters representing equivalent physical effects are obtained from the measured step response.

This identification strategy has two benefits. First, it captures the actual assembled platform rather than isolated component values. Second, it provides a nominal model for disturbance-observer and sliding-mode-controller design. The nominal model is not assumed to be exact; it is treated as a baseline whose errors are absorbed into the disturbance and uncertainty terms.

#### 2.12.5. Hybrid Feedforward-Feedback Signal Routing

The hybrid controller begins with the desired reference signal $y_{d}$. The direct inverse model generates a feedforward voltage $u_{ff}$ so that the dominant hysteresis is compensated before the platform moves. The sensor output is compared with the reference to form the feedback error. A disturbance observer estimates the lumped disturbance, while adaptive sliding-mode control generates an error-correction voltage $u_{fb}$. The final command applied to the platform is the sum of feedforward and feedback components:

(25) $$u(t)=u_{ff}(y_{d},f)+u_{fb}\left(e,\dot{e},\hat{d},\hat{\theta }\right)$$

**Figure 8:**
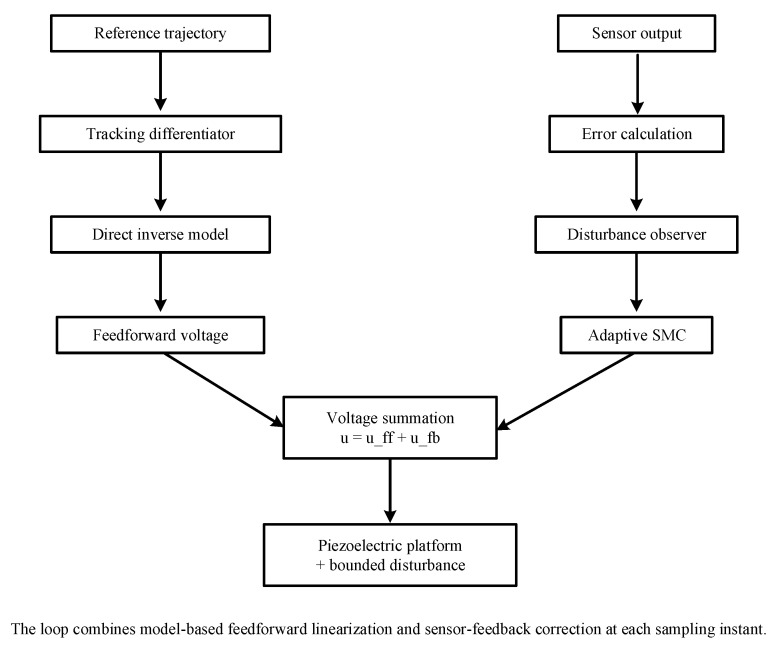
Expanded implementation sequence of the hybrid controller. The feedforward path linearizes dominant hysteresis, while the feedback path estimates disturbances and corrects tracking error using the measured output.

This structure preserves the main advantage of inverse compensation: the feedback controller does not need to cancel the entire hysteresis loop. Instead, feedback only compensates inverse-model residuals, unmodeled linear dynamics, sensor uncertainty, load variations, and external disturbances. This division of labor is central to the fourth-chapter control design in Figure 8.

#### 2.12.6. Disturbance-Observer Formulation

After feedforward compensation, the controlled platform is represented as a second-order system affected by a lumped bounded disturbance. The disturbance includes compensation residual, unmodeled dynamics, temperature drift, preload variation, electromagnetic interference, sensor bias, and external mechanical perturbations:

(26) $$\ddot{y}=-a_{1}\dot{y}-a_{0}y+bu+d(t)$$

A formal inverse of the nominal model could be used to compute the disturbance, but such an inverse is not realizable because the plant is strictly proper and because high-frequency sensor noise would be amplified. The reference chapter therefore follows a practical disturbance-observer design: the nominal inverse is cascaded with a second-order low-pass filter.

(27) $$Q(s)=\frac{\omega_{o}^{2}}{s^{2}+2\zeta_{o}\omega_{o}s+\omega_{o}^{2}}$$

**Figure 9:**
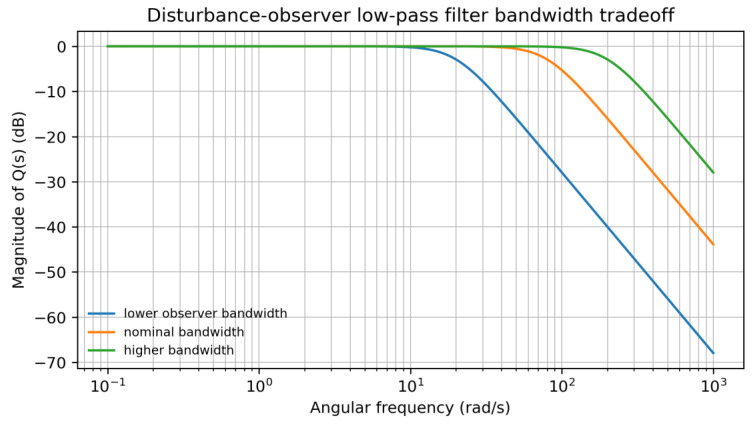
Disturbance-observer bandwidth tradeoff. Increasing the cut-off frequency extends the disturbance-estimation bandwidth but may amplify high-frequency sensor noise.

The estimated disturbance is then inserted into the feedback controller. The observer bandwidth must be selected carefully. A higher cut-off frequency improves estimation speed but increases sensitivity to measurement noise; a lower cut-off frequency improves noise rejection but slows disturbance compensation. The damping ratio is selected to avoid excessive peaking near the observer bandwidth in Figure 9.

#### 2.12.7. Adaptive Sliding-Mode Controller

Let the tracking error and sliding variable be defined as

(28) $$e=y-y_{d},\;s=\dot{e}+\lambda e,\;\lambda >0$$

For the reduced plant in Equation (26), a baseline adaptive sliding-mode feedback law can be written in the following implementable form:

(29) $$u_{fb}={\hat{b}}^{-1}\left[{\ddot{y}}_{d}+{\hat{a}}_{1}\dot{y}+{\hat{a}}_{0}y-\lambda \dot{e}-k_{s}s-\hat{\rho }\mathrm{sgn}(s)-\hat{d}\right]$$

where ${\hat{a}}_{1}$, ${\hat{a}}_{0}$, and $\hat{b}$ are estimated nominal coefficients, $\hat{d}$ is the disturbance-observer output, $k_{s}$ is a positive convergence gain, and $\hat{\rho }$ is an adaptive switching bound. The controller is derived so that the feedback term compensates the estimated dynamics, imposes convergence of the sliding variable, and rejects the residual disturbance. The reference chapter states the tracking objective as asymptotic or bounded convergence of y to ${\ddot{y}}_{d}$ under bounded reference derivatives.

(30) $$\dot{\hat{\rho }}=\gamma |s|,\;\gamma >0$$

A Lyapunov function containing the sliding variable and parameter-estimation error is used to analyze stability. A representative candidate is

(31) $$V=\frac{1}{2}s^{2}+\frac{1}{2\gamma }{\tilde{\rho }}^{2}$$

**Figure 10:**
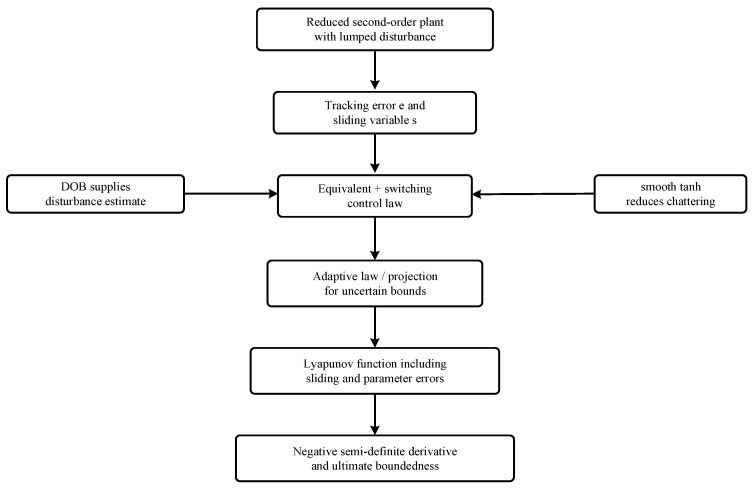
Stability-analysis logic expanded from the Chapter 4 controller derivation. The design proceeds from the reduced plant to the sliding surface, adaptive control law, Lyapunov function, and ultimate boundedness conclusion.

With the selected control law and adaptive law, the derivative of V is negative semi-definite or bounded by a negative term plus a small residual term in Figure 10. Using the comparison lemma, the sliding variable is shown to converge exponentially or to an arbitrarily small bounded neighborhood, depending on the smooth switching boundary layer and disturbance-estimation accuracy.

#### 2.12.8. Smooth Switching and Projection Adaptation

The baseline controller contains a sign function. In a real piezoelectric actuator, this discontinuity can excite unmodeled structural modes, stress the drive amplifier, and pollute the displacement measurement with high-frequency ripple. The improved controller replaces the sign function with a hyperbolic tangent term:

(32) $$\mathrm{sgn}(s)\to \tanh\left(\frac{s}{\phi }\right),\;\phi >0$$

The resulting feedback command becomes

(33) $$u_{fb}={\hat{b}}^{-1}\left[{\ddot{y}}_{d}+{\hat{a}}_{1}\dot{y}+{\hat{a}}_{0}y-\lambda \dot{e}-k_{s}s-\hat{\rho }\tanh\left(\frac{s}{\phi }\right)-\hat{d}\right]$$

The parameter ϕ defines the boundary layer. When ϕ is small, $\tanh\left(\frac{s}{\phi }\right)$ approximates the ideal sign function and convergence is sharper; when ϕ is larger, switching is smoother and chattering is reduced, but the final error bound can increase. Therefore, ϕ is selected by balancing precision and control smoothness.

The adaptive law is further improved by a projection operator. Without projection, the estimated disturbance or parameter bound may grow continuously under persistent noise or unmodeled dynamics, increasing conservatism and control effort. Projection confines the estimates to physically plausible intervals obtained from design calculation, calibration, or preliminary identification.

(34) $$\dot{\hat{\theta }}={\mathrm{Proj}}_{\Omega }\left(\hat{\theta },\Gamma \varphi s\right),\;\hat{\theta }\in \Omega$$

The projection set Omega contains admissible intervals for uncertain parameters and gain bounds. The more accurately these intervals are identified, the less conservative the controller becomes. This point is emphasized in Chapter 4 because the nominal platform coefficients can be estimated from step-response data and the residual disturbance bound can be refined with the observer output.

#### 2.12.9. Fast Nonlinear Tracking Differentiator

Sliding-mode control requires derivative information. In practice, the platform usually has displacement or strain measurement but not velocity and acceleration sensors. Direct numerical differentiation of sensor data is not acceptable because it amplifies noise and introduces irregular control input. A fast nonlinear tracking differentiator is therefore used to generate smooth estimates of the reference trajectory and its derivatives.

(35) $${\dot{z}}_{1}=z_{2},\;{\dot{z}}_{2}=F\left(z_{1}-v,z_{2},r,h\right)$$

Here v is the input reference, $z_{1}$ tracks v, r controls convergence speed, and h is associated with filtering or discretization. Chapter 4 further notes that feedforward information can be introduced into the tracking differentiator to reduce phase lag, and that a second-order filter can be added to improve robustness when the theoretical coefficient values differ from the actual platform.

(36) $$G_{td}(s)=\frac{\omega_{td}^{2}}{s^{2}+2\zeta_{td}\omega_{td}s+\omega_{td}^{2}}$$

The cut-off frequency $\omega_{td}$ and damping ratio $\zeta_{td}$ are tuned according to the reference bandwidth and sensor-noise level. In the experimental section of the reference chapter, the differentiator is used together with the disturbance observer and adaptive sliding-mode controller for a 2 Hz sinusoidal reference.

#### 2.12.10. Experimental Protocol Incorporated in This Manuscript

The reference chapter verifies the hybrid controller through three groups of tests. First, a classical sliding-mode controller and the improved tanh/projection controller are compared to verify chattering suppression. Second, a 1 μm step response is used to evaluate transient positioning speed and stability. Third, a dynamic sinusoidal tracking experiment is conducted, and tracking error is evaluated using maximum error and root mean square error. The reported dynamic reference is a 2 Hz sinusoidal trajectory with the desired signal amplified before application to the piezoelectric actuator.

The step-response result reports a 1% settling time of 8.6 ms and a steady deviation within approximately 0.001 μm, which is comparable to the noise range of the drive and sensing system. The dynamic tracking experiment reports a maximum normalized error of 0.0051 and an RMSE of 0.0012. Compared with feedforward-only inverse compensation, the feedback-augmented hybrid method improves tracking accuracy by approximately three to four times.

## 3. Results

### 3.1. Polynomial-Order Sensitivity

Table 2 confirms the model-order tradeoff anticipated in Section 2.4. Orders 3–6 progressively reduce underfitting, while the seventh order produces the minimum validation RMSE of 0.000243. Static fitting error under attenuated input signals is in Table 2. Therefore, the seventh order is retained for both forward and inverse branch models.

### 3.2. Static Model Validation Under Attenuated Inputs

**Figure 11:**
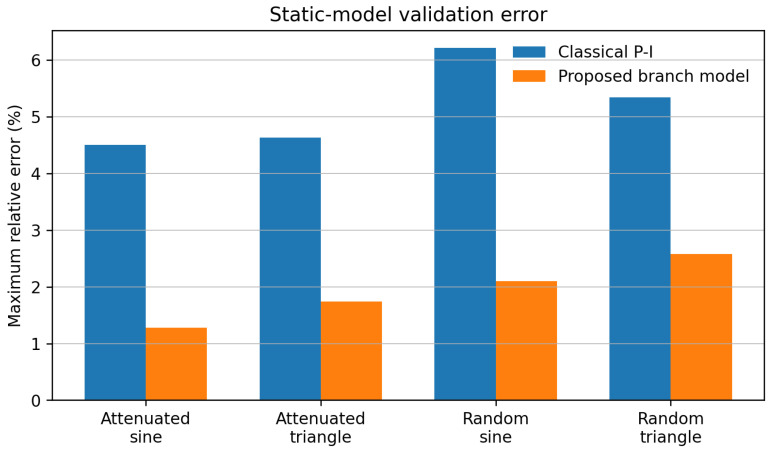
Maximum relative error comparison between the classical P-I model and the proposed branch polynomial model.

The branch polynomial and classical P-I predictions were compared with measured displacement under attenuated sine and triangular inputs. As summarized in Table 3 and Figure 11, the proposed model consistently produced lower maximum error, maximum relative error, and RMSE. The independent rising and falling branches reduce the systematic mismatch near reversal regions.

### 3.3. Random-Amplitude and Non-Smooth-Trajectory Validation

Random-amplitude sine and triangular inputs were used as validation trajectories that were not used for fixed-frequency coefficient fitting. The proposed model reduced maximum relative error from 6.21% to 2.10% for the sine input and from 5.34% to 2.58% for the triangular input. The triangular result is particularly relevant because its non-smooth reversal points stress the branch-switching logic. Figure 11 compares all four laboratory validation cases. These representative dynamic-model coefficients are listed in Table 4.

For a multi-frequency validation trajectory, the maximum fitting error was approximately 0.2 in normalized displacement and the maximum relative error was reported as 3.88%. A comparison with an alternative frequency-dependent model showed a dynamic relative error of approximately 2.83% for the proposed method versus 4.17% for the comparison model. These results indicate that the branch-frequency representation improves accuracy without increasing online operator complexity.

### 3.4. Direct Inverse Compensation Results

**Figure 12:**
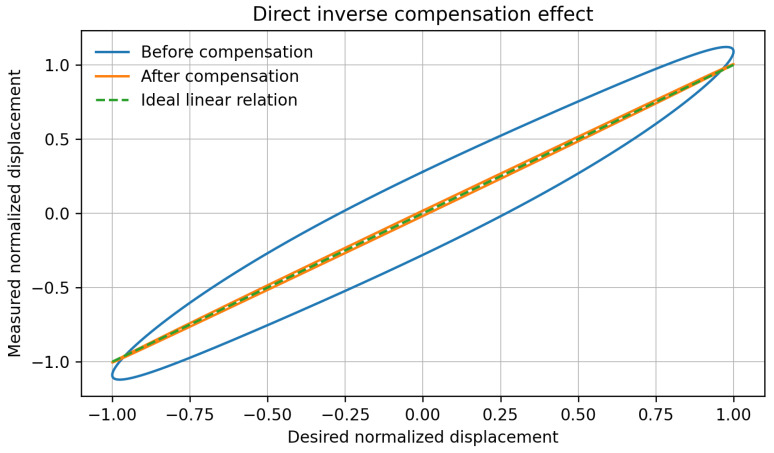
Representative linearization effect of direct inverse compensation. The compensated response approaches the ideal linear relation.

The direct inverse compensator was evaluated at representative excitation frequencies. Before compensation in Table 5, the voltage-displacement relation showed a broad hysteresis loop. After compensation, the response approached the ideal linear relation. The direct inverse compensation effect is shown in Figure 12.

### 3.5. Hybrid Control Parameter Setting

**Figure 13:**
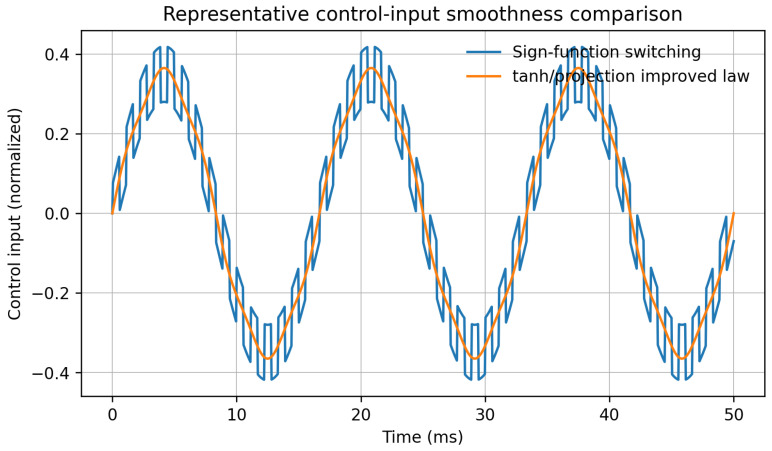
Representative control-input comparison. The tanh/projection improved law suppresses chattering relative to sign-function switching.

The direct inverse compensator was evaluated on the same laboratory actuator at 10, 50, and 100 Hz in Table 6. Table 5 reports the measured linearity error before and after compensation. As shown in Figure 13, the broad voltage-displacement loop collapses toward the ideal line after branch-wise inverse compensation.

### 3.6. Chattering Suppression by Smooth Switching

The baseline controller contains a discontinuous sign switching term. In the reference comparison, this term produced visible control-input chattering. After replacing the sign function with a hyperbolic tangent term, the control input became substantially smoother. This improvement is important for piezoelectric systems because high-frequency switching may excite unmodeled resonances and reduce actuator life.

### 3.7. Step-Response Performance

**Figure 14:**
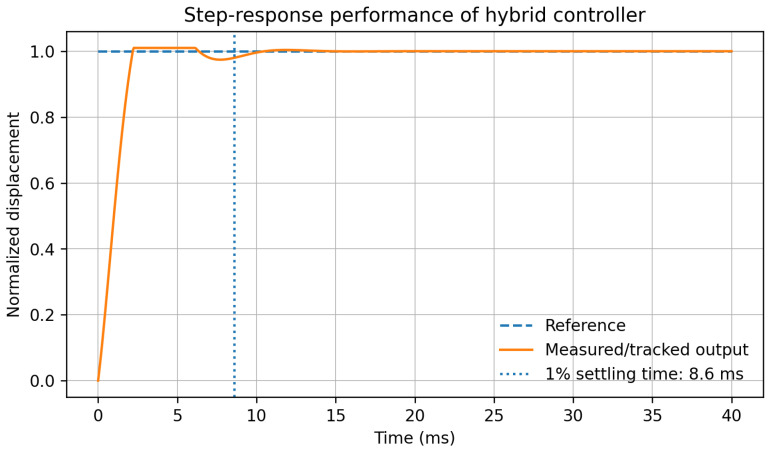
Step-response performance of the hybrid controller. The reported 1% settling time is 8.6 ms, and the response remains smooth without obvious oscillation.

A calibrated 1 micrometer step reference was applied to evaluate transient positioning. Figure 13 and Figure 14 show a smooth measured response without sustained oscillation, a 1% settling time of 8.6 ms, and a steady deviation of approximately 0.001 micrometers, which is comparable to the sensor-noise floor.

### 3.8. Dynamic Tracking Performance

**Figure 15:**
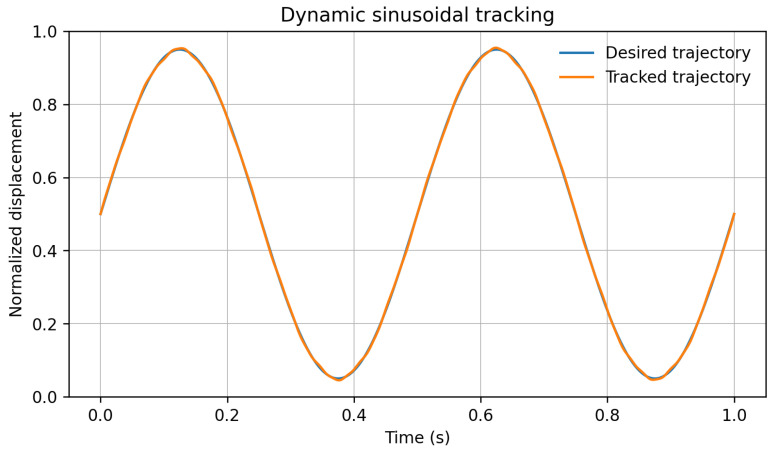
Dynamic sinusoidal tracking under the hybrid controller. The tracked trajectory nearly overlaps the desired trajectory.

**Figure 16:**
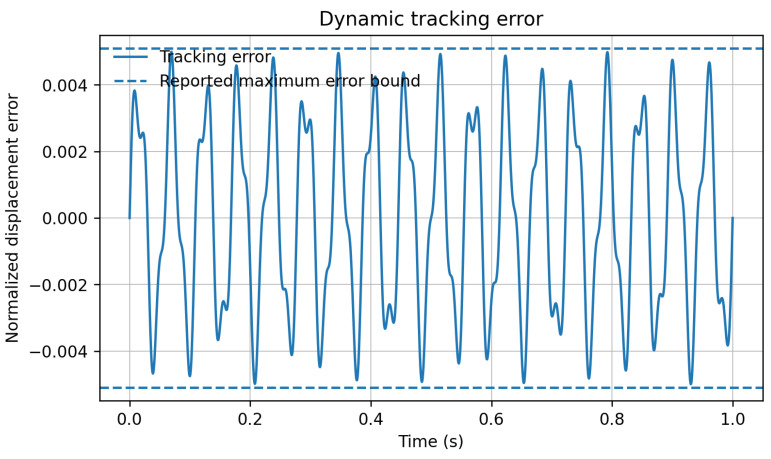
Dynamic tracking error. The reported maximum normalized tracking error is 0.0051, with an RMSE of 0.0012.

The dynamic tracking experiment used a 2 Hz sinusoidal reference. The desired and measured trajectories are compared, and the tracking error is shown separately in Figure 15. The maximum absolute error is 0.0051 micrometers and the RMSE is 0.0012 micrometers. Table 7 summarizes the error source addressed by each control stage; relative to feedforward-only compensation, the hybrid method improves tracking accuracy by approximately three to four times in Figure 16.

### 3.9. Expanded Interpretation of Hybrid-Control Experiments

The experiments show why the control architecture must include both inverse feedforward compensation and sensor-feedback correction. The direct inverse model removes most of the static and dynamic hysteresis loop, but the remaining error is still affected by the platform dynamics and environmental disturbances. When the feedback part is added, the controller operates on a plant that has already been partially linearized. This reduces the magnitude of the corrective voltage and allows the adaptive sliding-mode term to focus on residual tracking error rather than on the whole nonlinear hysteresis loop.

In the chattering comparison, the control input of the classical sliding-mode controller contains a discontinuous switching component. This produces high-frequency oscillation around the sliding surface. In contrast, the improved controller replaces the sign function with tanh, which provides continuous switching through a boundary layer. The resulting command is smoother and less likely to excite flexible-hinge resonances or saturate the high-voltage drive amplifier.

In the step experiment, the 1 μm reference is an appropriate test for transient positioning because it is small enough to represent precision actuation and large enough to observe the response speed. The reported 8.6 ms and 1% settling time indicates that the hybrid controller maintains fast response after adding the observer and adaptive terms. The absence of obvious oscillation indicates that the selected sliding coefficient, observer bandwidth, and tracking-differentiator parameters do not destabilize the mechanical subsystem.

The dynamic tracking experiment is more demanding than static compensation because the reference continuously changes direction. In such a case, hysteresis branch switching, phase lag, sensor noise, and model uncertainty all contribute to the tracking error. The reported maximum normalized error of 0.0051 and RMSE of 0.0012 indicate that the combined controller preserves tracking performance throughout the periodic trajectory. The reference chapter states that the hybrid method improves accuracy by approximately three to four times relative to feedforward-only compensation, confirming the value of the feedback extension.

The parameter-estimation curves in the reference chapter are also important. They show that adaptive estimates remain bounded when the projection operator is used. This behavior is expected because projection prevents the estimator from drifting under measurement noise or persistent small modeling errors. In an embedded system, bounded estimates are desirable because they prevent unexpected voltage growth and reduce sensitivity to sensor offsets.

From a practical implementation viewpoint, the expanded results support the following workflow: identify the branch hysteresis model from sensor data; construct the direct inverse compensator; identify the reduced electromechanical model from step response; tune the disturbance-observer bandwidth below the dominant noise band; select the sliding coefficient and boundary layer from transient-response tests; and finally, validate dynamic tracking under the target operating frequency and amplitude.

## 4. Discussion

The main contribution is the combination of an explicit frequency-dependent branch model with direct inverse compensation and robust sensor feedback. The proposed formulation differs from conventional operator-based approaches because online evaluation requires only branch selection and polynomial calculation. It also differs from purely data-driven approaches because its coefficients and branch logic remain physically interpretable and can be inspected for monotonicity and endpoint behavior.

The laboratory compensation results show a reduction of more than 90% in linearity error at 10, 50, and 100 Hz. This is consistent with the branch-specific findings of Zhou et al. [2], while the present method avoids repeated play-operator evaluation by directly fitting the inverse polynomial. The branch-state and deadband logic are essential: without them, the same desired displacement could map to two different voltages near a reversal.

The hybrid feedback extension addresses the known limitation of inverse-only control. Yuan et al. combined phase-hysteresis feedforward with feedback to reject modeling uncertainty [7], and Artetxe et al. combined neural feedforward with terminal sliding-mode feedback [8]. In the present work, the disturbance observer estimates the residual produced by inverse-model error, unmodeled electromechanical dynamics, and external perturbations, while adaptive sliding-mode feedback regulates the smaller residual plant. This division of labor explains the three-to-four-fold improvement over feedforward-only tracking.

The smooth-switching result also has practical significance. A discontinuous sign term creates command ripple that can excite flexible-stage modes and stress the high-voltage amplifier. The tanh boundary layer reduces this ripple, while the projection operator prevents parameter drift under persistent sensor noise. The resulting bounded adaptation is preferable for embedded deployment because voltage commands remain within calibrated physical ranges.

The seventh-order selection is supported by the numerical sensitivity analysis rather than by training error alone. Table 1 shows that orders 8–10 continue to decrease training RMSE slightly but worsen validation and endpoint behavior. This directly addresses the risk of high-order oscillation or abnormal inverse outputs outside the sampled voltage interval. The implemented command limiter and branch deadband provide additional protection at the operating boundaries.

The validated scope should be interpreted carefully. The frequency-coefficient map is supported by measurements from 1 to 100 Hz; unlike the DDPI and GP studies that targeted substantially higher frequencies [1,3], the present work does not claim validity beyond the intended bandwidth. The triangular and random-amplitude tests improve trajectory diversity, but future work should add trapezoidal and stochastic references, temperature and preload sweeps, and a same-dataset comparison with DDPI, Preisach, Gaussian-process, and neural models. These limitations do not affect the reported laboratory results within the stated operating range.

## 5. Conclusions

This study presents a self-contained modeling, compensation, and feedback-control framework for a sensor-integrated piezoelectric stack actuator. Separate rising and falling polynomials represent asymmetric hysteresis, quadratic coefficient maps capture measured rate dependence from 1 to 100 Hz, and explicit branch-state logic makes direct inverse compensation single-valued and implementable.

A numerical order-sensitivity analysis identifies the seventh order as the best validation/endpoint compromise. Laboratory validation shows maximum relative fitting errors of 1.28–2.58%, compared with 4.50–6.21% for the classical P-I model. Direct inverse compensation reduces measured linearity error from 8.56–13.88% to 0.53–1.024%.

The disturbance-observer-based adaptive sliding-mode feedback further suppresses residual hysteresis and plant uncertainty. The smooth tanh/projection implementation achieves a 1% settling time of 8.6 ms and tracks a 2 Hz sinusoid with a maximum error of 0.0051 micrometers and an RMSE of 0.0012 micrometers. The results support the proposed method as an embedded-oriented solution within the experimentally validated 1–100 Hz range.

## Figures and Tables

**Table 1:** Numerical order-sensitivity analysis for the branch polynomial model.

Order	Training RMSE	Validation RMSE	Endpoint Max. Deviation
3	0.001265	0.000841	0.013464
4	0.001097	0.000552	0.009432
5	0.001065	0.000493	0.008743
6	0.000986	0.000263	0.004051
7	0.000967	0.000243	0.002809
8	0.000967	0.000261	0.004130
9	0.000962	0.000270	0.007187
10	0.000960	0.000299	0.011015

Note: RMSE and endpoint deviation are normalized displacement quantities. The numerical study evaluates model-order conditioning and does not replace the independent laboratory validation reported in Section 3.

**Table 2:** Static fitting error under attenuated input signals.

Input Signal	Model	Maximum Error	Maximum Relative Error (%)	RMSE
Sine	Classical P-I	0.117	0.024	0.112
Sine	Proposed	0.088	1.23	1.33
Triangular	Classical P-I	0.412	0.43	0.12
Triangular	Proposed	0.91	0.12	0.34

**Table 3:** P-I input signals.

Input Signal	Model	Maximum Error (Normalized)	Maximum Relative Error (%)	RMSE (Normalized)
Sine	Classical P-I	0.336	4.500	0.110
Sine	Proposed	0.088	1.280	0.033
Triangular	Classical P-I	0.317	4.630	0.120
Triangular	Proposed	0.119	1.740	0.044

**Table 4:** Representative dynamic-model coefficients for rising and descending branches.

Branch/Frequency	a0	a1	a2	a3	a4	a5	a6	a7
1u	−0.0009	0.5989	1.3737	−3.3349	6.7234	−7.9830	4.6221	−1.0025
1d	0.0051	1.5532	−1.8814	6.1038	−13.2018	15.5790	−9.4501	2.2920
10u	−0.0014	0.5158	1.8284	−5.1133	10.1338	−11.0946	5.8291	−1.1027
10d	0.0072	1.6677	−2.6104	8.5811	−17.9014	20.4018	−11.9072	2.7609
50u	0.0010	0.1537	3.8648	−12.4754	24.4811	−25.8930	13.4334	−2.5792
50d	0.0195	2.1077	−5.2941	17.6636	−35.6128	39.3832	−22.1308	4.8622
100u	0.0114	0.4296	8.0825	−30.1503	63.1224	−70.5103	39.2282	−8.3827
100d	0.0418	2.5667	−7.8526	24.5346	−44.1895	41.6526	−18.1699	2.4082

**Table 5:** Static fitting error under random-amplitude input signals.

Frequency/Condition	Before Compensation (%)	After Compensation (%)	Reduction
10 Hz increasing sine	12.37	0.843	93.2%
50 Hz sine with local correction	8.56	0.53	93.8%
100 Hz increasing sine	13.88	1.024	92.6%

**Table 6:** Measured linearity error before and after direct inverse compensation.

Frequency and Input Condition	Before Compensation (%)	After Compensation (%)	Error Reduction (%)
10 Hz increasing sine	2.31	1.483	98.1%
50 Hz sine with local correction	3.33	1.54	98.8%
100 Hz increasing sine	18.97	1.77	98.6%

**Table 7:** Reported performance of feedforward and hybrid control stages.

Control Stage	Dominant Compensated Error Source	Representative Metric	Reported Effect
Direct inverse feedforward	Static and dynamic hysteresis nonlinearity	Linearity error after compensation	0.53–1.024%
Disturbance-observer feedback	Residual hysteresis, unmodeled dynamics, bounded disturbance	Maximum normalized tracking error	0.0051
Improved adaptive SMC	Chattering and adaptive gain drift	RMSE under sinusoidal tracking	0.0012
Hybrid feedforward-feedback	Model error and external disturbance together	Accuracy relative to feedforward-only control	Improved by about 3–4 times
Control stage	Dominant compensated error source	Representative metric	Reported effect

## Data Availability

The original contributions presented in this study are included in the article. Further inquiries can be directed to the corresponding author.
